# Reconstructing Life with Chronic Kidney Disease in Contexts of Health Inequality: A Grounded Theory Developed in Chile

**DOI:** 10.3390/ijerph23080996

**Published:** 2026-07-29

**Authors:** Mirliana Ramírez-Pereira

**Affiliations:** Department of Nursing, University of Chile, Avda. Independencia 1027, Santiago 8380453, Chile; mirliana@uchile.cl

**Keywords:** chronic kidney disease, dialysis, nursing theory, social determinants of health, health equity

## Abstract

**Highlights:**

**Public Health Relevance—How does this work relate to a public health issue?**

- Chronic kidney disease is a growing global public health challenge that disproportionately affects socially vulnerable populations and contributes substantially to morbidity, mortality, reduced quality of life, and healthcare utilization.
- This study examines how people living with chronic kidney disease on long-term dialysis progressively reconstruct their lives, highlighting the influence of health inequities, social determinants of health, and healthcare experiences throughout this process.

**Public Health Significance—Why is this work of significance to public health?**

- This study develops a substantive theory explaining how life reconstruction is sustained through the dynamic interaction between resignation and survival and facilitated by disease understanding, social support networks, and nursing care.
- The findings demonstrate that opportunities for life reconstruction depend not only on individual agency but also on the availability of supportive social and healthcare conditions.

**Public Health Implications—What are the key implications or messages for practitioners, policy makers and/or researchers in public health?**

- Public health policies and healthcare services should strengthen the facilitating conditions that support life reconstruction, including health education, person-centred nursing care, continuity of care, and access to social support resources.
- Efforts to reduce health inequities should recognize that living well with chronic kidney disease depends on both individual capacities and equitable access to social, community, and healthcare resources that enable long-term well-being.

**Abstract:**

Chronic kidney disease (CKD) is a major public health challenge associated with substantial physical, psychological, social, and economic consequences. Although its clinical outcomes have been extensively investigated, less is known about how individuals progressively reconstruct their lives while living with an irreversible condition requiring long-term dialysis, particularly in contexts characterized by health inequities. This study aimed to develop a substantive theory explaining how adults reconstruct their lives while living with CKD on dialysis. A qualitative study was conducted using Strauss and Corbin’s Grounded Theory methodology. Eighteen adults receiving hemodialysis or peritoneal dialysis in Chile participated in semi-structured interviews. Data collection and analysis were conducted concurrently using theoretical sampling, the constant comparative method, memo writing, and theoretical saturation. The findings identified “Reconstructing Life While Living with Chronic Kidney Disease” as the central phenomenon. Life reconstruction emerged as a dynamic and ongoing process sustained by the interaction between resignation and survival and facilitated by three interrelated conditions: understanding the disease, social support networks, and nursing care. Through this process, participants progressively reorganized their identities, everyday lives, interpersonal relationships, and future life projects while living with an irreversible chronic condition. The resulting substantive theory provides a contextually grounded explanation of life reconstruction that integrates individual, relational, and social dimensions of living with CKD. By demonstrating that opportunities for life reconstruction are shaped not only by individual agency but also by supportive social and healthcare conditions, the findings contribute to nursing knowledge and provide a conceptual foundation for person-centered care, health equity, and future research on chronic illness.

## 1. Introduction

Chronic kidney disease (CKD) is defined by abnormalities of kidney structure or function that persist for at least three months and have implications for health. Its progression may lead to kidney failure requiring dialysis or kidney transplantation. CKD is closely associated with diabetes mellitus, hypertension, and cardiovascular disease and represents a major and growing cause of morbidity, disability, and premature mortality worldwide [1].

Over the past few decades, the global burden of CKD has increased steadily. The Global Burden of Disease study has identified CKD as one of the fastest-growing causes of death worldwide, and it is estimated to affect approximately 10% of the adult population. Owing to its progressive nature, CKD generates substantial health, social, and economic consequences, including increased use of healthcare resources, reduced quality of life, hospitalization, disability, cardiovascular events, and premature mortality [2,3].

Beyond its clinical burden, CKD also represents an important health-equity challenge. Its incidence, progression, access to treatment, and outcomes are unequally distributed across social groups. People experiencing socioeconomic vulnerability, limited educational opportunities, restricted access to healthcare services, unstable employment, or territorial barriers to specialized care may face greater risks of disease progression and fewer opportunities to maintain well-being. These inequalities reflect the influence of social determinants of health on the prevention, diagnosis, treatment, and outcomes of CKD [2,3,4,5].

In Latin America, these inequalities are reinforced by structural limitations associated with fragmented and segmented health systems. Opportunities for prevention, early diagnosis, specialized care, and kidney replacement therapy remain unevenly distributed across and within countries. Regional evidence has shown substantial differences in access to dialysis, transplantation, specialized human resources, and healthcare financing, disproportionately affecting socially disadvantaged populations [6,7].

The regional situation is further shaped by population ageing, the increasing prevalence of diabetes mellitus and hypertension, and persistent inequalities in access to healthcare services. Data from the Latin American Dialysis and Renal Transplant Registry indicate a sustained increase in the number of people requiring kidney replacement therapy, although treatment availability and coverage continue to vary considerably between countries [1,3,8,9,10,11].

Chile has one of the highest prevalence rates of kidney replacement therapy in Latin America. National records show sustained growth in the population receiving chronic dialysis. Between 2012 and 2022, the number of people undergoing chronic hemodialysis increased by 41.9%, from 17,014 to 24,139, and national prevalence has continued to rise in subsequent years [12].

Chile also has an extensive public financing system for kidney replacement therapy. Advanced CKD and kidney failure are included within the national system of Explicit Health Guarantees, which provides access to specialist care, dialysis, kidney transplantation, and follow-up services. For beneficiaries of the National Health Fund, hemodialysis and peritoneal dialysis are provided with financial protection, reducing direct treatment costs for most people receiving these therapies [12].

However, financial coverage of dialysis does not eliminate the broader social and personal consequences of living with CKD. Dependence on long-term treatment makes the interaction between individual and structural conditions particularly visible. Family support, employment stability, social protection, access to specialized services, transportation, educational resources, and the quality of healthcare relationships may either support or constrain people’s opportunities to reconstruct everyday life and maintain a dignified life [10,13,14,15].

People living with advanced CKD also face multiple challenges associated with dietary and fluid restrictions, physical limitations, work-related difficulties, changes in family roles, altered social participation, and disruption of future life plans [13,14,15,16]. These experiences extend beyond the biological consequences of kidney dysfunction and encompass psychological, relational, cultural, economic, and spiritual dimensions. CKD should therefore be understood not only as an organic disorder but also as a complex human experience through which people construct meanings, reorganize their everyday lives, and seek ways to live with an irreversible condition [13].

Previous qualitative studies have documented the physical symptoms, emotional distress, treatment burden, loss of autonomy, altered family roles, employment difficulties, and restrictions in everyday life experienced by people receiving dialysis [17,18,19]. This body of research has made an important contribution by identifying patients’ perceptions, support needs, and coping experiences. Nevertheless, much of the existing literature remains primarily descriptive or organized around thematic findings.

Less is known about how the different dimensions of the experience interact over time and how people reconstruct continuity, meaning, identity, and future life projects while living with an irreversible condition requiring long-term dialysis. In addition, theoretical explanations grounded in the experiences of people living in Latin American contexts marked by social, territorial, and healthcare inequalities remain limited.

Addressing this gap is important because adaptation to CKD cannot be understood solely as an individual psychological response. The possibilities of reorganizing everyday life may also depend on access to social support, nursing care, healthcare resources, employment protection, and broader structural conditions. A contextually grounded theory may therefore contribute to nursing knowledge by explaining how personal, relational, clinical, and social dimensions interact in the process of living with CKD.

Accordingly, this study aimed to develop a substantive grounded theory explaining how adults receiving dialysis reconstruct their lives while living with chronic kidney disease in a context shaped by social and health inequalities.

## 2. Materials and Methods

### 2.1. Study Design

A qualitative study was conducted using the grounded theory approach developed by Strauss and Corbin, under a post-positivism paradigm. This approach was selected because it enables the systematic generation of an explanatory theory grounded in participants’ experiences and in the relationships identified through concurrent data collection and analysis. The study sought to develop a contextually situated explanation of how people receiving dialysis reconstruct their lives while living with CKD [20,21].

### 2.2. Researcher Characteristics and Reflexivity

The interviews and primary data analysis were conducted by the first author, a nurse researcher with clinical experience in nephrology and formal training in qualitative research. The researcher had no previous therapeutic or personal relationship with the participants and was not involved in their clinical care.

Her professional background facilitated familiarity with the clinical context, terminology, and care processes, while also acknowledging that prior clinical experience could influence assumptions regarding illness experiences, self-care, treatment adherence, and nursing practice.

To enhance reflexivity throughout the study, reflexive notes were recorded following each interview and during the analytical process. These notes documented methodological decisions, emerging interpretations, personal reflections, and the evolution of categories. Throughout the analysis, the researcher repeatedly returned to the original transcripts and applied constant comparison to ensure that the emerging categories and theoretical explanations were grounded in participants’ accounts rather than pre-existing professional assumptions.

### 2.3. Participants and Context

The participants were adults diagnosed with chronic kidney disease who were receiving kidney replacement therapy through hemodialysis or peritoneal dialysis.

Eligible participants were adults aged 18 years or older who had been diagnosed with chronic kidney disease, were receiving maintenance hemodialysis or peritoneal dialysis, were able to communicate their experiences, and were willing to provide written informed consent. Individuals presenting with acute kidney injury, cognitive impairment or clinical conditions that prevented participation in an in-depth interview, current hospitalization, clinical instability, or inability to provide informed consent were excluded from the study. Eligibility was initially assessed by a designated healthcare professional at each participating dialysis center and subsequently confirmed by the researcher before informed consent was obtained.

Initial participants were selected purposively to obtain variation in age, sex, dialysis modality, and time receiving treatment. As data collection and analysis progressed concurrently, preliminary coding informed subsequent recruitment decisions. For example, when early interviews suggested that treatment modality influenced autonomy, body image, and the organization of everyday life, additional participants receiving peritoneal dialysis and hemodialysis were recruited to explore similarities and differences between these experiences. Likewise, emerging findings concerning family dependence and social support led to the inclusion of participants with different marital, household, and employment circumstances. Sampling continued until the main categories were sufficiently developed in terms of their properties, dimensions, and relationships.

Participants were recruited from dialysis centers located in Northern Chile. Both dialysis modalities were intentionally included to explore shared and potentially contrasting experiences of living with long-term kidney replacement therapy.

Theoretical saturation was assessed based on the absence of new conceptual properties, dimensions, or relationships emerging from the data rather than on the simple repetition of participants’ accounts. Saturation was reached with participant 17; however, one additional interview was conducted to assess the stability and explanatory consistency of the emerging categories [21]. The final sample consisted of 18 participants (Table 1).

### 2.4. Data Collection

Data was collected between April 2016 and January 2017. A total of 18 individual semi-structured interviews were conducted, one with each participant. No follow-up interviews were undertaken, as the concurrent processes of data collection and analysis provided sufficient depth to develop the emerging categories and achieve theoretical saturation.

The semi-structured interview guide was developed based on study objectives and relevant literature [22]. Prior to data collection, the guide was piloted with one individual who met the study eligibility criteria to assess the clarity, relevance, and sequencing of the questions. Minor refinements were made to improve the wording and flow of the interview guide before commencing data collection. Data obtained during the pilot interview was not included in the final analysis.

Interviews lasted between 40 and 88 min, with an average duration of approximately 64 min. All interviews were conducted face-to-face in private rooms within the participating dialysis units, ensuring privacy, confidentiality, and a comfortable environment for participants.

The interviews were guided by broad, open-ended questions designed to explore participants’ experiences of living with chronic kidney disease and receiving kidney replacement therapy. Consistent with the principles of Grounded Theory, data collection and analysis occurred concurrently, allowing the interview guide to evolve as concepts emerged from the data. Additional questions and probes were incorporated throughout the study to further explore developing categories, their properties, dimensions, and relationships.

All interviews were conducted in Spanish, audio-recorded with participants’ written informed consent, and transcribed verbatim. Field notes were recorded immediately after each interview to document contextual observations, non-verbal interactions, preliminary analytical reflections, and emerging theoretical insights that informed subsequent data collection and analysis.

Quotations selected for publication were translated from Spanish into English by a professional translator. The translated quotations were subsequently reviewed against the original Spanish transcripts by the principal investigator to ensure semantic accuracy, preserve participants’ intended meanings, and maintain conceptual equivalence between the original and translated versions.

### 2.5. Data Analysis

Data analysis followed the systematic procedures of Grounded Theory described by Strauss and Corbin [21]. Data collection and analysis were conducted concurrently, allowing emerging findings to inform theoretical sampling and the refinement of subsequent interviews through the constant comparative method.

All transcripts were coded and analyzed by the first author. Because coding was performed by a single researcher, intercoder agreement was not applicable. To enhance analytic rigor, an audit trail was maintained throughout the study to document coding decisions, methodological decisions, category development, revisions to the coding framework, and the progressive integration of the emerging theoretical model. Throughout the analytical process, categories were continuously compared within and across interviews and repeatedly examined against the original data to ensure conceptual consistency and theoretical integration.

Each transcript was read repeatedly to achieve a comprehensive understanding of the participants’ narratives before formal coding began. Open coding was then conducted through a line-by-line examination of the data to identify concepts and assign codes representing participants’ experiences, actions, and meanings. The qualitative data were analyzed using ATLAS.ti 8.0 for Windows (Scientific Software Development GmbH, Berlin, Germany). This software was used to organize, manage, and retrieve the qualitative data throughout the analytical process.

During axial coding, relationships among categories and subcategories were established by examining the conditions, actions/interactions, and consequences associated with the phenomenon under study. This stage facilitated a deeper understanding of the contextual factors shaping participants’ experiences of chronic kidney disease, the meanings they attributed to their illness, and the strategies they developed to cope with everyday life.

Finally, selective coding was undertaken to integrate the categories into a coherent explanatory framework by identifying the core category and systematically relating all categories to it. Throughout this stage, analytic memos were used to document theoretical insights, explore relationships among categories, refine their properties and dimensions, and support the integration of the substantive grounded theory.

The analytical process resulted in the identification of 546 initial codes, which were progressively grouped into 13 categories. Through successive stages of abstraction and integration, these categories were organized into four major conceptual families: understanding the illness, social support networks, consequences of the illness, and nursing care. The integration of these conceptual families led to the development of a substantive grounded theory explaining the process through which individuals reconstruct their lives while living with chronic kidney disease.

### 2.6. Rigor and Trustworthiness

The rigor of the study was established according to the criteria of credibility, transferability, dependability, confirmability, and theoretical-epistemological coherence [23].

Credibility was enhanced through concurrent data collection and analysis, theoretical sampling, constant comparison, and the progressive refinement of categories until theoretical saturation was achieved.

Transferability was supported by providing a detailed description of the study context, participant characteristics, and methodological procedures, enabling readers to assess the applicability of the findings to similar contexts.

Dependability was strengthened through the maintenance of an audit trail documenting methodological decisions, coding procedures, category development, and the evolution of the emerging theoretical model.

Confirmability was promoted through reflexive notes, analytic memos, and systematic documentation of analytical decisions, ensuring that interpretations remained grounded in participants’ accounts.

Theoretical-epistemological coherence was maintained by ensuring consistency between the philosophical assumptions of Grounded Theory, the study design, data collection, analysis, and the development of substantive theory.

Although member checking and formal peer debriefing were not undertaken, methodological rigor was ensured through the combined use of theoretical sampling, the constant comparative method, reflexive documentation, analytic memos, maintenance of an audit trail, and the concurrent processes of data collection and analysis, all of which are consistent with the methodological principles of Grounded Theory as described by Strauss and Corbin.

### 2.7. Ethical Considerations

This study was conducted in accordance with the ethical principles of the Declaration of Helsinki, including respect for autonomy, beneficence, non-maleficence, and justice [24].

All participants received verbal and written information about the study objectives, participation procedures, possible risks and benefits, and the voluntary nature of their participation. Subsequently, they signed an informed consent form before the interview began.

Anonymity was guaranteed using alphanumeric codes and the removal of any data that could identify the participants or healthcare centers involved. Participants were informed that they could withdraw from the study at any time without consequences for their clinical care.

Audio recordings and transcripts were stored on password-protected devices, with access restricted exclusively to the research team members.

This study was approved by the Scientific Ethics Committee in Nursing (CECENF) at Universidad Andrés Bello—approval number: L2/CECENF.

## 3. Results

### 3.1. Open Coding

In the first stage of the analysis, open coding was performed. Thirteen categories emerged from the interviews, which were subsequently grouped into four conceptual families: understanding of the disease, social support networks, consequences of the disease, and nursing care. These categories made it possible to understand the meanings attributed by individuals to chronic kidney disease and to the experience of living with dialysis.

#### 3.1.1. Friend Category

This category describes the relationships participants maintained with their friends after the diagnosis of chronic kidney disease. Initially, many participants concealed their condition because they feared pity, discrimination, or losing their previous identity, which contributed to feelings of isolation and reduced social interaction. Over time, however, some participants developed supportive relationships with other dialysis patients who shared similar experiences and understood the challenges associated with the disease. These relationships became an important source of emotional support and a sense of belonging.

*“My friends don’t even know that I’m sick, I don’t tell them, because for me illnesses are more or less personal things, here there are two kinds of attitudes towards illness, there are people who say everything they feel, they say it out loud, I don’t say anything because I think nobody cares about what I have”*.

(B.G.)

#### 3.1.2. Healthcare Team Category

This category represents participants’ experiences with the multidisciplinary healthcare team involved in their treatment. Its central dimensions were professional competence, continuity of care, team stability, and trust developed over time. Unlike the relational qualities described in the Warmth in Care category, this category focuses on the role and perceived expertise of the healthcare team.

*“The professionals at the dialysis center are specialized people, very good, people who understand the subject… here I have found a clinic that knows their stuff, they’re not improvising, not leading you down another path… they’re people who know”*.

(A.M.)

#### 3.1.3. Spirituality and Illness Category

This category encompasses the spiritual meanings associated with chronic kidney disease. Participants described God, faith, and religious participation as fundamental resources for coping with the disease. Spirituality was associated with feelings of refuge, hope, and comfort, as well as with interpretations of the illness as a divine punishment or a spiritual trial. Participation in religious communities also provided emotional support, a sense of belonging, and new ways of understanding the experience of illness.

*“That God sent it to me, it must be for a reason. Well, God doesn’t want to make you suffer; it’s your own fault… something you haven’t done right. I wonder if there isn’t anything I’ve done wrong… maybe I haven’t taken care of myself. God wants you to take care of yourself, to dedicate yourself to Him. You dedicate yourself to your home and leave God aside”*.

(B.G.)

#### 3.1.4. Family Category

Families emerged as one of the main sources of support for people receiving dialysis. Participants described the practical and emotional support provided by children, partners, siblings, and other relatives. This support was expressed through accompaniment, assistance with dialysis-related tasks, supervision of care, and emotional support. However, participants also described tensions associated with bodily changes, particularly among those undergoing peritoneal dialysis who reported feelings of shame and altered self-image.

*“…I feel supported… at first, they did not want me to exert myself… not even pick up a chair. They are surprised and wonder how I can be so active with this illness”*.

(C.R.)

#### 3.1.5. Healthcare System Category

This category included participants’ experiences with health services and chronic kidney disease care programs. Participants described distrust toward some healthcare facilities, difficulties in obtaining a timely diagnosis, and delayed initiation of renal replacement therapy. They also recognized the importance of funding mechanisms and health coverage in facilitating access to treatment.

*“They told me it would take a little longer, and I would fall into a coma. I was so intoxicated, so ill. I was like that for about three days, and then they started dialysis, inserting the catheter. All I could do was cry and cry”*.

(C.V.)

#### 3.1.6. Physical Consequences of the Illness Category

This category describes the physical effects of chronic kidney disease and renal replacement therapies. Participants reported complications such as infections, anemia, hypotension, cramps, and persistent physical limitations. They also expressed concern about the possibility of death and the bodily changes associated with the disease and its treatment.

*“My blood pressure would drop, I got home in really bad shape, I spent more time in bed than on my feet, I couldn’t do anything; they had to help me with everything, it was very hard”*.

(A.C.).

#### 3.1.7. Category: Day-to-Day Restrictions

This category represents the limitations imposed by chronic kidney disease and its treatment on participants’ daily lives. Participants described dietary restrictions, limitations on travel, difficulties with sexual activity, reduced physical activity, and a loss of autonomy in decisions related to their treatment. Dependence on dialysis was experienced as a loss of personal freedom that restricted everyday life.

*“…It’s a burden I carry because I can’t even go out anymore. I think about going out and the machine, about not having dialyzed. What’s the point of traveling if I must travel with the same machine? It’s not the same as being at home, I can’t”*.

(V.C.)

#### 3.1.8. Category: Psychosocial Consequences

This category describes the emotional, social, and economic impacts of chronic kidney disease. Participants reported workplace discrimination, fear of abandonment, financial difficulties, loss of community participation, and significant changes in their life plans. The illness profoundly affected their personal identity and future expectations.

*“I am retired, but I do some consulting, on a smaller scale, a few gigs, because people already see you as half sick, so they give you less work. This affects me because this disease is expensive; there are medicines the doctor prescribes, but they are not available at the clinic. Being on dialysis is not cheap”*.

(R.A.)

#### 3.1.9. Category: Self-Care in the Health-Illness Continuum

This category includes the actions participants took to maintain their health, prevent complications, and adhere to treatment. Participants described a gradual learning process about the disease, dietary recommendations, dialysis techniques, and measures to prevent complications. Self-care was perceived as a fundamental strategy for maintaining clinical stability and preserving continuity in everyday life.

*“I must follow the doctor’s procedure, all their instructions; I do not have any other option. I have learned a bit about this disease. I like to learn, for example, about creatinine, that wicked thing that causes you to end up on dialysis. It’s a heavy mineral the kidney does not remove anymore; maybe it takes out very little, but once you are on dialysis there’s no going back”*.

(N.C.)

#### 3.1.10. Category: Understanding the Causes of Kidney Disease

This category describes the experiences associated with hemodialysis and peritoneal dialysis. Participants acknowledged these therapies as essential for survival while simultaneously experiencing them as a source of dependence and limitation. They also emphasized the importance of trusting the healthcare team and adopting preventive measures to avoid treatment-related complications.

*“It was more my own neglect. I worked a lot, had seven kids, and did not have time to take care of myself. I had a baby and, as soon as I got out of the maternity ward, I was washing, ironing, and doing everything at home. I never took care of myself, and I think that’s why…”*.

(R.P.)

#### 3.1.11. Category: Renal Replacement Therapies

This category describes the experiences associated with hemodialysis and peritoneal dialysis.

Participants acknowledged the importance of these therapies for survival, while simultaneously perceiving them as a source of dependence and limitation. Trust in healthcare staff and the prevention of complications stand out as central elements of the therapeutic experience.

Associated codes: nursing experience, trust in the healthcare team, infection prevention, and dependence.

*“If I do it as they tell me, I go to all my checkups, I walk. To avoid infections, I am very careful; I wash my hands, use a sanitizer, and have a clean area where the machine is. I like to keep my things tidy and clean”*.

(D.C.)

This category describes the experiences associated with hemodialysis and peritoneal dialysis. Participants acknowledged renal replacement therapies as essential for survival, recognizing that continuing treatment was the only way to remain alive. At the same time, they described a gradual process of resignation, expressed through the acceptance of lifelong treatment, dependence on dialysis, and the loss of freedom imposed by the disease. Renal replacement therapy was therefore experienced not only as a life-sustaining treatment but also as a continuous process of adapting to a new way of life.

*“…one feel tied down. Before, I could just go to Viña or Santiago whenever I wanted; I had more freedom. Now I feel tied down… I must accept what the Lord has determined and continue living. I cannot rebel, because if I stop dialysis for a month, I die.”*.

(A.M.)

#### 3.1.12. Category: Warmth in Care

This category represents the interpersonal and emotional qualities expressed during care encounters. Participants valued closeness, empathy, kindness, availability, and genuine concern, while also describing distress when care was delivered with indifference or hostility. Unlike the Healthcare Team Category, its focus is not on professional roles or technical competence, but on how participants felt treated in everyday interactions. Participants contrasted positive experiences characterized by empathy and closeness with situations in which they perceived indifference or disrespect in their interactions with healthcare professionals.

*“The nurse, she’s a sweetheart, I’ll say that right away, she’s nice and she greets you and cares about us”*.

(D.M.)

*“Sometimes there are nurses who come to work in a bad mood. Sometimes, they treat patients poorly. One time, I was treated badly, and I left and did not get dialyzed that day. They are there to work, not to bring their anger from home and take it out on patients. We’re the sick ones”*.

(L.G.)

#### 3.1.13. Category: Ethics in Care

This category reflects participants’ evaluations of the ethical conduct of healthcare professionals during the provision of care. Responsibility, honesty, respect, accountability, and commitment to patient safety were central to their evaluations. Unlike the Warmth in Care category, which concerns the interpersonal quality of care, this category reflects participants’ expectations that healthcare professionals act responsibly, acknowledge mistakes, communicate transparently, and prioritize patient safety.

*“It was a nurse’s negligence; she left the line open, and the blood was coming back. She disconnected three patients simultaneously. I asked her for an explanation, and she denied everything. No one told me what had happened, and about 20 days later, I had to receive a blood transfusion.”*.

(D.M.)

### 3.2. Higher-Order Categories

The constant comparative process identified conceptual relationships among the categories developed during open coding. Through progressive abstraction, the 13 initial categories were integrated into four higher-order categories representing central dimensions of the experience of living with CKD. This integration supported the transition from descriptive categories to an explanatory model of the phenomenon.

#### 3.2.1. Metacategory 1 Understanding the Disease

This metacategory emerged from the integration of the categories Understanding the Causes of Kidney Disease, Self-care in the Health–Illness Continuum, and Renal Replacement Therapies.

Together, these categories explain how participants progressively constructed an understanding of chronic kidney disease and its treatment. Initially, they sought to explain the causes of kidney deterioration, frequently attributing it to inadequate self-care, work demands, family responsibilities, or spiritual beliefs. As they gained experience with the disease, participants acquired knowledge about dialysis, treatment requirements, and self-care practices, enabling them to better manage their condition and actively participate in their own care.

Renal replacement therapy became a turning point in this process of understanding. Participants recognized dialysis as indispensable for survival while simultaneously describing a gradual process of resignation, expressed through the acceptance of lifelong treatment, dependence on dialysis, and the loss of freedom imposed by the disease. Consequently, understanding the disease extended beyond acquiring information; it became a process of making sense of an irreversible condition and adapting to a new way of life.

This progressive understanding reduced uncertainty, strengthened self-care, and laid the foundation for the subsequent reconstruction of everyday life.

#### 3.2.2. Metacategory 2 Social Support Networks

This metacategory emerged from the integration of the categories Family, Friends, and Spirituality and Illness.

Participants experienced chronic kidney disease within a network of supportive relationships that influenced their ability to cope with the disease and its consequences. Family members provided practical assistance, emotional support, and accompaniment throughout the treatment process. Friends, particularly other people receiving dialysis, contributed understanding, companionship, and a sense of belonging that reduced feelings of isolation. Spirituality provided hope, comfort, and meaning, allowing participants to reinterpret difficult experiences and maintain emotional strength in the face of uncertainty.

Taken together, these social support networks buffered the emotional and social impact of chronic kidney disease, facilitated adaptation, and strengthened participants’ capacity to reconstruct their lives despite the limitations imposed by the illness.

#### 3.2.3. Metacategory 3 Consequences of Disease

This metacategory emerged from the integration of the categories Physical Consequences of the Illness, Day-to-day Restrictions, Psychosocial Consequences, and Healthcare System.

Together, these categories illustrate the multidimensional consequences of chronic kidney disease in participants’ lives. The illness affected not only physical health through symptoms, treatment-related complications, and functional limitations, but also emotional well-being, employment, family relationships, social participation, and future life plans. Participants described profound changes in their daily routines, increasing dependence on treatment, and restrictions that progressively limited their autonomy.

These experiences were also shaped by participants’ interactions with the healthcare system. Access to timely diagnosis, continuity of care, treatment opportunities, and healthcare resources influenced how the consequences of the disease were experienced and managed. Thus, the impact of chronic kidney disease resulted not only from its biological progression but also from the healthcare context in which participants lived and received treatment.

Together, these consequences constituted the context within which participants were required to reorganize their everyday lives and progressively adapt to an irreversible condition.

#### 3.2.4. Metacategory 4 Nursing Care

This metacategory emerged from the integration of the categories Healthcare Team, Warmth in Care, and Ethics in Care.

Participants described nursing care as a multidimensional experience that combined professional competence, interpersonal relationships, and ethical practice. Confidence in the healthcare team was built through professional expertise, continuity of care, and the perception that healthcare professionals possessed the knowledge and skills required to manage complex treatments. At the interpersonal level, participants highly valued warmth, empathy, kindness, availability, and genuine concern, which fostered trust and emotional security throughout the illness trajectory. Ethical practice, expressed through responsibility, honesty, transparency, accountability, and commitment to patient safety, reinforced participants’ confidence in the care they received and shaped their evaluations of professional conduct.

Together, these dimensions positioned nursing care as a fundamental therapeutic resource that promotes trust, safety, emotional support, and adaptation throughout the process of living with chronic kidney disease and long-term dialysis.

### 3.3. Axial Coding

Axial coding enabled the integration of the categories developed during open coding into an explanatory framework that described the dynamic process through which participants reconstructed their lives while living with chronic kidney disease. Rather than representing isolated experiences, the categories became analytically connected through relationships among causal conditions, contextual factors, intervening conditions, action–interaction strategies, and consequences. This integration revealed that participants’ experiences evolved through a continuous process of life reconstruction in response to an irreversible illness.

The process was initiated by the diagnosis of chronic kidney disease and the progressive deterioration of renal function. Participants described the onset of persistent symptoms, repeated hospitalizations, uncertainty regarding the future, and the realization that kidney failure represented a permanent condition. The initiation of renal replacement therapy constituted a major turning point, transforming the imminent threat of death into the necessity of lifelong treatment. Previous personal experiences, family histories of chronic illness, and participants’ interpretations of the causes of kidney disease further shaped the meanings attributed to the diagnosis and influenced their initial responses.

Life reconstruction unfolded within a context characterized by the multidimensional consequences of chronic kidney disease. Physical symptoms, functional limitations, emotional distress, restrictions in daily activities, changes in family roles, reduced social participation, and alterations in future life plans profoundly affected participants’ everyday lives. These experiences were further influenced by the healthcare system, including access to timely diagnosis, continuity of care, availability of treatment, and the quality of healthcare services. Consequently, participants’ life reconstruction was shaped not only by the progression of the disease itself but also by the broader healthcare context in which they received care.

The central phenomenon identified through axial coding was Reconstructing Life While Living with Chronic Kidney Disease. Rather than representing a single adaptive event, this phenomenon described a continuous process through which participants reorganized their identities, daily routines, interpersonal relationships, and future expectations following the disruption caused by an irreversible illness.

The analytical integration revealed that this reconstruction process was driven by the dynamic interaction between survival and resignation. Survival reflected participants’ determination to preserve life despite the physical, emotional, and social demands imposed by chronic kidney disease and dialysis treatment. Resignation, in contrast, represented the gradual acceptance of the irreversible nature of the disease, lifelong dependence on renal replacement therapy, and the loss of freedom associated with treatment. Rather than functioning as opposing responses, survival and resignation operated as complementary processes that continuously shaped participants’ interpretations, decisions, and life reconstruction throughout the illness trajectory. Together, they explained how participants gradually transformed the initial disruption caused by the diagnosis into a reconstructed way of living.

This reconstruction process was facilitated by several intervening conditions. As participants progressively understood the disease, its causes, treatment, and self-care requirements, uncertainty diminished, and their ability to actively participate in their care increased. Family members, friends, and spirituality constituted essential sources of emotional, practical, and existential support, helping participants cope with the demands of chronic illness. Similarly, nursing care emerged as a fundamental facilitating condition. Professional competence, continuity of care, interpersonal warmth, and ethical practice fostered trust, emotional security, confidence in treatment, and participants’ willingness to engage in long-term care.

In response to these conditions, the participants developed multiple action–interaction strategies aimed at preserving life while adapting to an irreversible condition. These strategies included adhering to dialysis treatment, incorporating self-care behaviors into daily life, modifying dietary habits, monitoring symptoms, seeking information about the disease, relying on family and social support, engaging in spiritual practices, and reorganizing everyday routines according to the limitations imposed by treatment. Through these adaptive actions, participants progressively re-established a sense of normality despite the permanence of chronic kidney disease.

**Figure 1:**
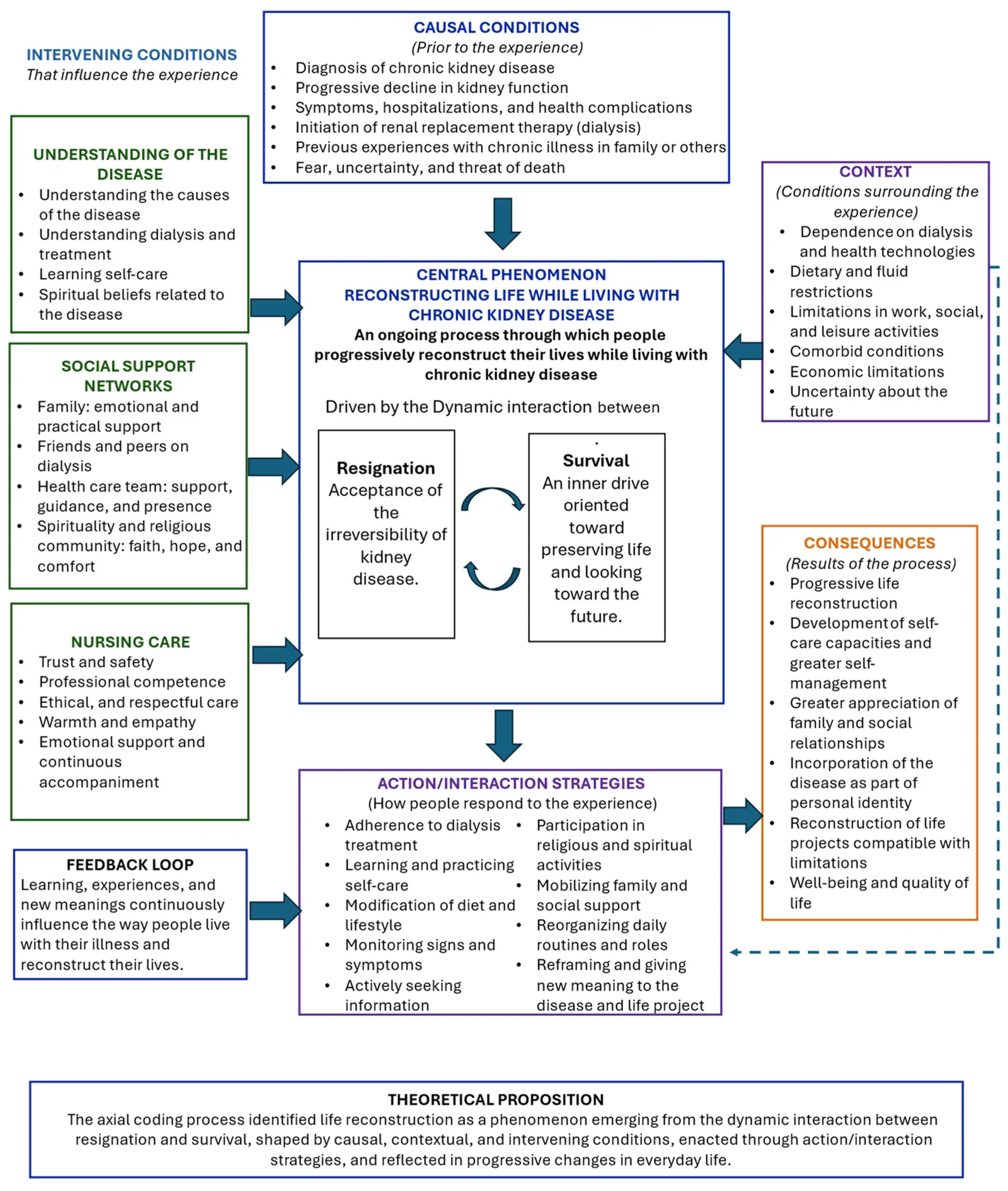
Axial Model of Reconstructing Life While Living with Chronic Kidney Disease on Dialysis. Source: Developed by the author from the study findings. Note: Solid arrows indicate the direction of conceptual relationships, circular arrows represent the dynamic interaction between resignation and survival, and the dashed arrow indicates the feedback process. These relationships are theoretical and do not imply causality.

Because of this process, participants gradually reconstructed their lives by integrating chronic kidney disease into their personal identities, redefining their priorities, strengthening meaningful relationships, and developing new ways of living with the disease. Although physical limitations, emotional challenges, and dependence on treatment persisted, participants increasingly described themselves as capable of living with chronic kidney disease rather than being completely defined by it. Life reconstruction, therefore, emerged as a dynamic, ongoing process sustained by the continuous interaction between survival and resignation, providing the analytical foundation for the substantive theory developed in this study (Figure 1).

### 3.4. Substantive Theory of Life Reconstruction with Chronic Kidney Disease

The substantive theory developed in this study explains how people undergoing dialysis progressively reconstruct their lives while living with chronic kidney disease. Life reconstruction emerged as a dynamic and ongoing process through which individuals reorganize their identities, everyday routines, interpersonal relationships, and future life projects in response to an irreversible illness. Rather than occurring as a single adaptive event, reconstruction evolves continuously as individuals reinterpret their experiences and learn to live with the demands imposed by chronic kidney disease.

The analysis identified two interdependent processes that sustain life reconstruction: resignation and survival. Resignation refers to the progressive acceptance of the irreversible nature of chronic kidney disease and the incorporation of this reality into everyday life. Rather than representing passivity or surrender, resignation reflects an active process of acknowledging the permanence of the disease while adapting to its limitations. Survival represents the enduring motivation to preserve life, maintain meaningful relationships, and continue pursuing future goals despite the demands of dialysis and chronic illness. These processes operate simultaneously and dynamically, allowing individuals to reinterpret their experiences and progressively reconstruct their lives.

Life reconstruction is shaped by three facilitating conditions. Progressive understanding of the disease enables individuals to make sense of their condition, acquire self-care knowledge, and reduce uncertainty. Social support networks—including family members, friends, and spirituality—provide emotional, practical, and existential support that sustains adaptation throughout the illness trajectory. Nursing care, characterized by professional competence, interpersonal warmth, continuity, and ethical practice, promotes trust, emotional security, and confidence in long-term treatment. Together, these conditions strengthen individuals’ capacity to respond to the challenges associated with chronic kidney disease while preserving hope and meaningful life projects.

As individuals mobilize these facilitating conditions, they progressively reorganize their daily lives by incorporating self-care into everyday routines, redefining personal priorities, strengthening meaningful relationships, and integrating chronic kidney disease into their personal identities. Although illness remains a permanent part of life, it gradually ceases to be experienced exclusively as a threat and becomes a condition with which individuals learn to live. Consequently, life reconstruction is expressed through an ongoing process of adaptation that allows individuals to preserve purpose, dignity, and meaningful participation in family and social life despite the permanence of chronic kidney disease.

#### Central Theoretical Proposition

People living with chronic kidney disease undergoing dialysis progressively reconstruct their lives through the dynamic interaction between resignation and survival. This process is shaped by their understanding of the disease, social support networks, and nursing care, enabling them to reinterpret the experience of chronic illness and progressively reorganize their daily lives while preserving meaningful life projects.

**Figure 2:**
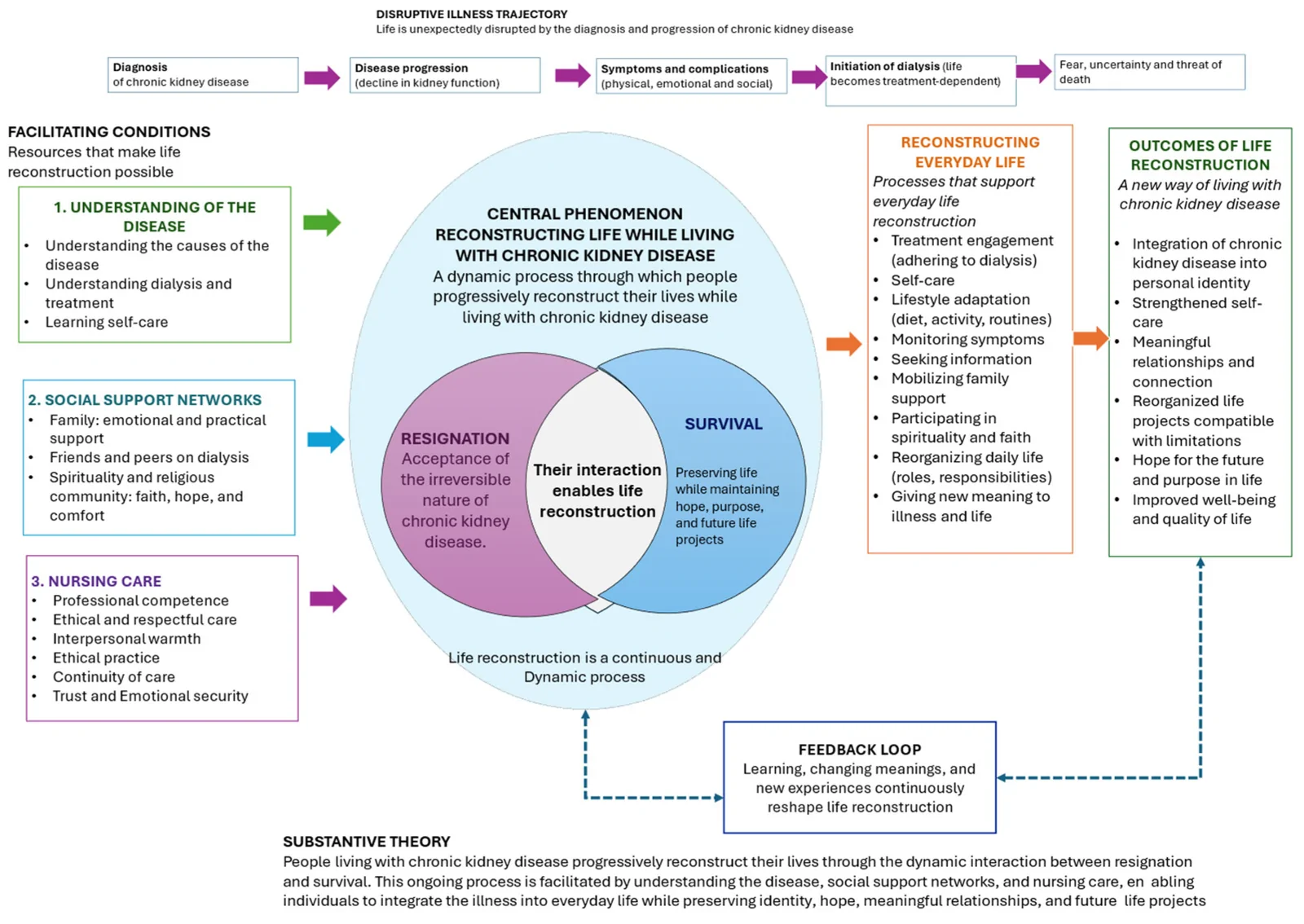
Substantive Theory of Reconstructing Life While Living with Chronic Kidney Disease on Dialysis. Source: Developed by the author from the study findings. Note: Solid arrows indicate the direction of the theoretical relationships among the illness trajectory, facilitating conditions, the central phenomenon, everyday life reconstruction, and its outcomes. Dashed arrows represent the feedback loop through which outcomes, learning, and new experiences reshape meanings and the ongoing reconstruction process. These relationships are conceptual and do not imply statistical causality.

This substantive theory offers an explanatory framework for understanding how individuals experience and respond to chronic kidney disease beyond its biomedical manifestations (Figure 2). The broader implications of this theoretical model for nursing practice, theory development, and future research are discussed in the following section (Figure 2).

## 4. Discussion

The present study developed a substantive theory that explains how individuals undergoing dialysis progressively reconstruct their lives while living with chronic kidney disease. Rather than conceptualizing adaptation as a linear response to illness, the theory proposes that life reconstruction is an ongoing and dynamic process sustained by the interaction between resignation and survival. This interaction is shaped by three facilitating conditions—understanding the disease, social support networks, and nursing care—which enable individuals to reorganize their daily lives, redefine their identities, and preserve meaningful life projects despite the permanence of chronic kidney disease.

The principal contribution of this theory lies in identifying life reconstruction as the central phenomenon through which individuals respond to chronic kidney disease. Existing nursing and chronic illness theories have described adaptation as a process of adjustment, transition, coping, or self-management [25]. In contrast, the present theory suggests that living with chronic kidney disease involves a broader process in which individuals progressively reconstruct their lives by integrating the illness into their identities while preserving purpose, dignity, and meaningful participation in family and social life. This reconstruction is neither linear nor definitive; rather, it evolves continuously as individuals reinterpret their experiences and respond to new challenges throughout the illness trajectory.

A central contribution of the theory is the conceptualization of resignation and survival as complementary and mutually reinforcing processes. Participants described resignation not as passivity or surrender but as the progressive recognition and acceptance of the irreversible nature of chronic kidney disease. Accepting the permanence of the illness allowed them to redirect their efforts from resisting the disease toward learning how to live with it. At the same time, survival emerged as the enduring motivation to preserve life, maintain meaningful relationships, pursue future aspirations, and continue participating in everyday life despite the physical, emotional, and social demands imposed by dialysis. Rather than representing opposing responses, resignation and survival coexist dynamically, constituting the central explanatory mechanism through which life reconstruction becomes possible.

These findings extend the Chronic Illness Trajectory Framework proposed by Corbin and Strauss, which conceptualizes chronic illness as a dynamic process requiring continuous adaptation, illness work, and the reorganization of everyday life involving individuals, families, and healthcare professionals. The present theory supports this perspective by demonstrating that life with chronic kidney disease is characterized by continuous adjustment rather than a single adaptive event. However, it contributes additional conceptual specificity by identifying the interaction between resignation and survival as the subjective mechanism through which individuals reconstruct their lives. Furthermore, dialysis is conceptualized not only as a medical treatment but also as a distinctive experience of technological dependence that profoundly shapes identity, everyday routines, and future life planning [21].

The theory also complements Meleis’s Transition Theory, which describes health–illness transitions as multidimensional processes involving changes in roles, relationships, identities, and patterns of daily living. Consistent with this perspective, participants experienced profound transitions from lives organized around autonomy to lives structured by treatment schedules, bodily limitations, and varying degrees of dependence. However, the present findings suggest that successful navigation of these transitions depends not only on individual adaptation but also on the availability of facilitating conditions, particularly disease understanding, supportive relationships, and nursing care that promotes confidence, continuity, and emotional security throughout the transition process [26,27].

Similarly, the findings resonate with Paterson’s Shifting Perspectives Model of Chronic Illness, which proposes that individuals alternate between illness-in-the-foreground and wellness-in-the-foreground perspectives. Participants in the present study also described fluctuating experiences in which the disease sometimes dominated everyday life and, at other times, became integrated into a broader sense of self. Nevertheless, the substantive theory extends Paterson’s model by explaining the process through which this integration occurs. Rather than describing shifts in perspective alone, the theory identifies the dynamic interaction between resignation and survival as the mechanism through which individuals progressively reconstruct meaningful lives while living with an irreversible chronic condition [28].

The findings also complement Wagner’s Chronic Care Model, particularly regarding the importance of informed patients, self-management, coordinated healthcare, and supportive community resources. However, whereas Wagner’s model primarily addresses the organization of health systems and service delivery, the present theory focuses on the lived experiences of individuals receiving dialysis. It therefore contributes a person-centred explanation of how people reconstruct their lives within healthcare systems, emphasizing that successful self-management depends not only on clinical interventions but also on relational, emotional, and social resources that support everyday living [29,30].

One of the distinctive contributions of the theory is its recognition of facilitating conditions as essential elements of life reconstruction. Progressive understanding of the disease enabled participants to reduce uncertainty, develop self-care competencies, and make informed decisions regarding treatment and daily living. Social support networks—including family members, friends, fellow patients, and spirituality—provided emotional, practical, and existential resources that sustained adaptation throughout the illness trajectory. Nursing care emerged as a particularly influential facilitating condition. Beyond technical competence, participants emphasized continuity, interpersonal warmth, ethical practice, trust, and emotional support as fundamental attributes that strengthened confidence and promoted active engagement in living with chronic kidney disease. These findings reinforce contemporary perspectives that position nursing as a relational practice in which technical expertise and human connection are inseparable components of high-quality care [13,14,15,16,31].

The theory also contributes to current discussions on health equity by demonstrating that life reconstruction occurs within broader social, economic, and healthcare contexts. Facilitating conditions are not equally available to all individuals. Access to education, financial security, family resources, employment opportunities, transportation, and specialized healthcare services directly influence individuals’ opportunities to understand the disease, mobilize support, and engage effectively in self-care. Consequently, life reconstruction cannot be understood solely as an individual achievement but must also be interpreted as a socially situated process shaped by structural opportunities and constraints. This perspective is particularly relevant in Latin America, where persistent social and territorial inequalities continue to influence access to chronic disease care and long-term health outcomes [6,7,8,9,32].

These findings have important implications for nursing practice. The theory suggests that nursing interventions should extend beyond symptom management and technical treatment to actively strengthen the facilitating conditions that support life reconstruction. Educational strategies should enhance individuals’ understanding of chronic kidney disease and promote confidence in self-care. Nursing interventions should also foster therapeutic relationships characterized by continuity, empathy, ethical commitment, and recognition of patients’ personal life projects. Likewise, nursing professionals can facilitate connections with family members, peer support, community resources, and spiritual care when appropriate, thereby strengthening the relational resources that sustain long-term adaptation [16,17,18,19,20,21,22,23,24,25].

The substantive theory developed in this study provides practical guidance for nursing practice, healthcare organizations, and public health policy by demonstrating that life reconstruction extends beyond clinical management and is shaped by the interaction between individual, relational, and structural conditions.

For nephrology nurses, the findings highlight the importance of promoting disease understanding through ongoing therapeutic education, strengthening person-centred therapeutic relationships, supporting self-care, and facilitating shared decision-making throughout the illness trajectory. Nursing care should also address the emotional, relational, and existential dimensions of chronic kidney disease, recognizing that life reconstruction is a dynamic process requiring continuous professional support.

For dialysis centres and healthcare organizations, the findings support the implementation of interdisciplinary models of care that strengthen continuity of care, psychosocial support, family involvement, and coordinated follow-up. These organizations should create care environments that foster trust, effective communication, and sustained therapeutic relationships, while ensuring that educational and supportive interventions are integrated into routine clinical practice rather than being limited to treatment initiation.

For public health authorities and policy makers, the findings suggest that strategies to reduce health inequities should extend beyond ensuring access to dialysis. Policies should also strengthen health literacy, improve access to community and family support resources, promote continuity across levels of care, and address the social determinants that influence people’s opportunities to reconstruct their lives while living with chronic kidney disease. Integrating these actions into chronic disease programs may contribute to more equitable, person-centred, and socially responsive models of care.

Although this substantive theory was developed within the Chilean healthcare context, its conceptual propositions may inform future research and the design of context-sensitive interventions in other settings characterized by social and health inequalities.

This study has several limitations. First, the substantive theory was developed from the experiences of individuals receiving dialysis in Chile and therefore reflects a specific sociocultural and healthcare context. Although the conceptual relationships identified may be relevant to other settings, their applicability should be explored through future qualitative research conducted in different countries, healthcare systems, and sociocultural contexts.

Another limitation is that participants receiving hemodialysis and peritoneal dialysis were analyzed within the same substantive theory because the study focused on the shared meanings and processes through which individuals reconstruct their lives while living with chronic kidney disease. Although common patterns emerged across both treatment modalities, some experiences appeared to be modality-specific, such as body-image concerns reported by participants undergoing peritoneal dialysis. Consequently, the integration of both groups may have obscured differences associated with the characteristics of each dialysis modality. Future studies could further explore these modality-specific experiences to refine and extend the substantive theory across different forms of kidney replacement therapy.

The perspectives of family caregivers and healthcare professionals were not included. Incorporating these perspectives in future studies could provide a more comprehensive understanding of the relational processes that shape life reconstruction.

Finally, all stages of data collection and analysis were conducted by a single researcher. Although methodological rigor was enhanced through theoretical sampling, the constant comparative method, analytic memo writing, reflexive documentation, and the maintenance of an audit trail, the absence of multiple analysts may have limited opportunities for interpretive triangulation. Future studies involving collaborative analysis or multidisciplinary research teams could further strengthen the refinement and validation of the emerging substantive theory.

## 5. Conclusions

In conclusion, this study offers a substantive theory that advances understanding of how individuals progressively reconstruct their lives while living with chronic kidney disease on dialysis. By identifying the dynamic interaction between resignation and survival and highlighting the role of disease understanding, social support networks, and nursing care as facilitating conditions, the theory provides a coherent explanation of life reconstruction that extends beyond traditional concepts of adaptation and coping. It also emphasizes that reconstruction is shaped not only by individual agency but also by the social and healthcare environments in which people live. As such, the theory offers a valuable conceptual foundation for nursing practice, future qualitative inquiry, and the development of interventions and policies aimed at strengthening the conditions that enable people to live well with chronic kidney disease.

## Figures and Tables

**Table 1:** Sociodemographic and clinical characteristics of participants (*N* = 18).

Participant	Gender	Age (Years)	Education	Occupation	Marital Status	Dialysis Modality	Time on Dialysis	Cause of Chronic Kidney Disease
A.R.	Male	72	Completed Secondary Education	Retired	Cohabiting	Hemodialysis	4 years	Hypertension
A.C.	Female	53	Completed Secondary Education	Homemaker	Separated	Hemodialysis	1 year and 1 month	Polycystic Kidney Disease
R.P.	Female	70	Primary Education	Retired	Widowed	Hemodialysis	1 year and 5 months	Type 2 Diabetes Mellitus
N.C.	Female	31	Completed Secondary Education	Homemaker	Cohabiting	Hemodialysis	11 years	Unknown
D.C.	Female	22	Higher Education	Student (Education)	Single	Peritoneal dialysis	6 years	Hypertension
B.G.	Male	77	Completed Secondary Education	Retired	Married	Peritoneal dialysis	5 years	Hypertension
C.V.	Female	46	Completed Secondary Education	Homemaker	Cohabiting	Hemodialysis	2 years	Fabry Disease
C.R.	Female	39	Higher Education	Translator and Interpreter	Married	Peritoneal dialysis	2 years and 8 months	Focal Segmental Glomerulosclerosis
V.C.	Female	80	Primary Education	Homemaker	Married	Peritoneal dialysis	6 years	Hypertension
D.M.	Male	41	Completed Secondary Education	School Inspector	Single	Hemodialysis	10 years	Loxoscelism
R.A.	Male	36	Higher Education	Commercial Engineer	Single	Hemodialysis	3 years	Unknown
A.M.	Male	65	Higher Education	Engineer	Married	Peritoneal dialysis	8 months	Diabetes Mellitus
W.A.	Male	54	Completed Secondary Education	Self-employed Merchant	Married	Hemodialysis	1 year	Obstructive Uropathy
E.Z.	Male	44	Completed Secondary Education	Taxi Driver	Single	Hemodialysis	2 years	Hypertension
J.L.	Male	53	Completed Secondary Education	Self-employed Merchant	Married	Hemodialysis	3 years	Hypertension
J.H.	Male	29	Completed Secondary Education	Electrician	Married	Hemodialysis	1 year	Congenital Chronic Kidney Disease
L.G.	Female	46	Completed Secondary Education	Administrative Assistant	Single	Hemodialysis	5 years	Hypertension
L.M.	Male	68	Primary Education	Farmer	Married	Hemodialysis	1 year	Hypertension

## Data Availability

The data presented in this study are not publicly available due to ethical and legal restrictions related to the protection of participants’ confidentiality and privacy under Chilean regulations governing research involving human subjects. De-identified data may be available from the corresponding author upon reasonable request and subject to approval by the relevant ethics requirements.
